# Probability Density Analysis Reveals Substantial Differences Between the Dinitrogen and Acetylene Triple Bonds

**DOI:** 10.1002/jcc.70037

**Published:** 2025-02-03

**Authors:** Michel V. Heinz, Emma Gorgas, Nicole Maser, Arne Lüchow

**Affiliations:** ^1^ Institute of Physical Chemistry RWTH Aachen University Aachen Germany

**Keywords:** chemical bonding, Lewis structures, probability density analysis, quantum Monte Carlo, wave function analysis

## Abstract

In earlier publications, it was shown that the electron positions maximizing the probability density ${\left|\Psi \right|}^{2}$ resemble the Lewis structures for most small molecules. While this holds for the triple bond in acetylene, this is not the case for the triple bond in dinitrogen. Because of recent advances in studying the topology of wave functions, this peculiar case is revisited. In this work, the dinitrogen wave function is analyzed and compared to that of acetylene. Significant differences of the electron positions maximizing ${\left|\Psi \right|}^{2}$ are uncovered and explained by the presence of hydrogen atoms in acetylene and by electron arrangements resulting from calculations of the nitrogen and carbon atoms. Moreover, insights into the electron delocalization of both molecules are gained by investigating electron exchange paths. Considering the different chemical behaviors of dinitrogen and acetylene, these differences should be expected.

## Graphical abstract

In recent years, the investigation of the many‐electron probability density ${\left|\Psi \right|}^{2}$ has made significant progress. Starting out, the electron positions locally maximizing ${\left|\Psi \right|}^{2}$ were studied and connected to Lewis structures. Subsequently, a measure of importance was defined for these structures as well as the exchange paths between these structures was investigated. This work uses this enlarged tool set to analyze the differences in topology of the dinitrogen and acetylene wave functions.

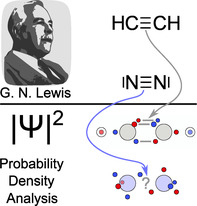

## 1 Introduction

The most important vocabulary of the chemical language are the Lewis structures, first described by G. N. Lewis in 1916 [1]. Nonetheless, connecting these sketches to the high‐dimensionality of modern quantum mechanical calculations has turned out to be difficult. Multiple methods have been developed over the years for investigating the chemical bond. In valence bond theory, for instance, a Lewis structure is represented by linear combination of Slater determinants. A weight can then be attributed to each structure as a measure of importance, giving insight into the nature of the bond [2]. Moreover, analyses based on molecular orbital theory, like the natural bond orbital theory [3] or the electron localization function [4], have grown popular. Also, the electronic density, a three‐dimensional function obtained by integrating the many‐electron probability density ${\left|\Psi \right|}^{2}$ over all but one electron, can be studied using Bader's quantum theory of atoms in molecules [5] (QTAIM). However, many‐electron effects will be lost as a consequence of this integration. In contrast to QTAIM, by analyzing the high‐dimensional probability density directly, the positions of all electrons can be evaluated simultaneously. This enables the study of many‐electron effects.

According to the Born rule, ${\left|\Psi \right|}^{2}$ is the probability density of the quantum particles which can, in principle, be measured, making it a physical observable. The discussion of the most probable electron arrangement, or maximum of the probability density, has a long history in the chemical literature [6, 7, 8]. Scemama et al. have first calculated maxima quantitatively, and one of the current authors has investigated the electron positions locally maximizing ${\left|\Psi \right|}^{2}$ for wave functions composed of Jastrow correlated Slater determinants for a set of small molecules [9, 10]. Since the many‐electron probability density was analyzed, the method was termed probability density analysis (PDA). A clear connection between the Lewis structures and the maxima positions of ${\left|\Psi \right|}^{2}$ was discovered allowing insight into electron structure complementary to the orbital or mean field picture. Most maxima can be rationalized solely by Coulomb interaction of electrons and nuclei and the Pauli repulsion. Pauli repulsion is not a force, but a result of the antisymmetry of the wavefunction, and its effect is visible in the maxima as core‐valence separation and larger distances for same‐spin electrons than for opposite‐spin electrons. Among other things, it was demonstrated, that the electron positions that maximize ${\left|\Psi \right|}^{2}$ for acetylene align with the Lewis structure of a triple bond. However, Schmidt and coworkers observed that examining the maxima of the dinitrogen wave function does not result in electron positions resembling the Lewis structures of a triple bond. Despite this, their method, based on tiling the wave function by means of Voronoi tessellation, reproduces the Lewis structure for dinitrogen [11].

Moreover, considering only the electron positions of the maxima of ${\left|\Psi \right|}^{2}$ does not include any information about the topology around it or its relevance to the complete wave function, which has been correctly pointed out by Scemama et al. [9]. Therefore, PDA was extended to not only consider the local maxima of the probability density, but also other topological features.

Similar to the critical points in QTAIM, we characterize in PDA the probability density ${\left|\Psi \right|}^{2}$ by its critical points in 3N‐dimensional space. Thus, PDA can be considered as a 3N‐dimensional analogue of QTAIM. In comparison to QTAIM, where the maxima of the electronic density contain information about the positions and nature of the nuclei, the local maxima of ${\left|\Psi \right|}^{2}$ are the locally most likely electron positions. Most of these maxima only differ in permutations of electrons, which are considered equal due to their indistinguishability. They are generated by maximizing the probability density for different starting electron positions taken from a sample, which is distributed according to ${\left|\Psi \right|}^{2}$. These arrangements are clustered together to eliminate their exponentially scaling number, thus simplifying their interpretation [12]. However, only studying the maxima does not suffice to describe the complete wave function. Therefore, they are interpreted as representative structures for PDA basins, which are similarly defined to the basins of attraction in QTAIM. By integrating the probability density for each basin, a measure of importance can be defined, termed PDA weight. By adding up the weights of all PDA structures the whole probability density is covered. Moreover, as long as the representative structure for each basin can be mapped to a Lewis structure, the PDA weights can be compared with weights from valence bond theory and a good correlation could be demonstrated [13, 14]. In addition to the electron positions maximizing the probability density, the paths connecting these maxima can be studied. For that purpose, a probability potential,

(1) $$\Phi =-\frac{1}{2}\ln{\left|\Psi \right|}^{2},$$

is defined, which changes the multiplicative nature of probabilities into an addition of potentials. As a result of the negative sign, maxima become minima, denoted structure critical points (SCPs), but the locations of the critical points remains the same. These minima are connected via first order saddle points, called delocalization critical points (DCPs), of the probability potential as they characterize the electron delocalization between two locally most probable electron arrangements. The paths connecting the minima via these saddle points are called maximum probability paths. Each path is associated with a probability barrier ΔΦ calculated by subtracting the probability potential of the reference minimum from the potential of the saddle point [15].

(2) $$\Delta \Phi =\Phi_{\mathrm{DCP}}-\Phi_{\mathrm{SCP}}$$

**FIGURE 1:**
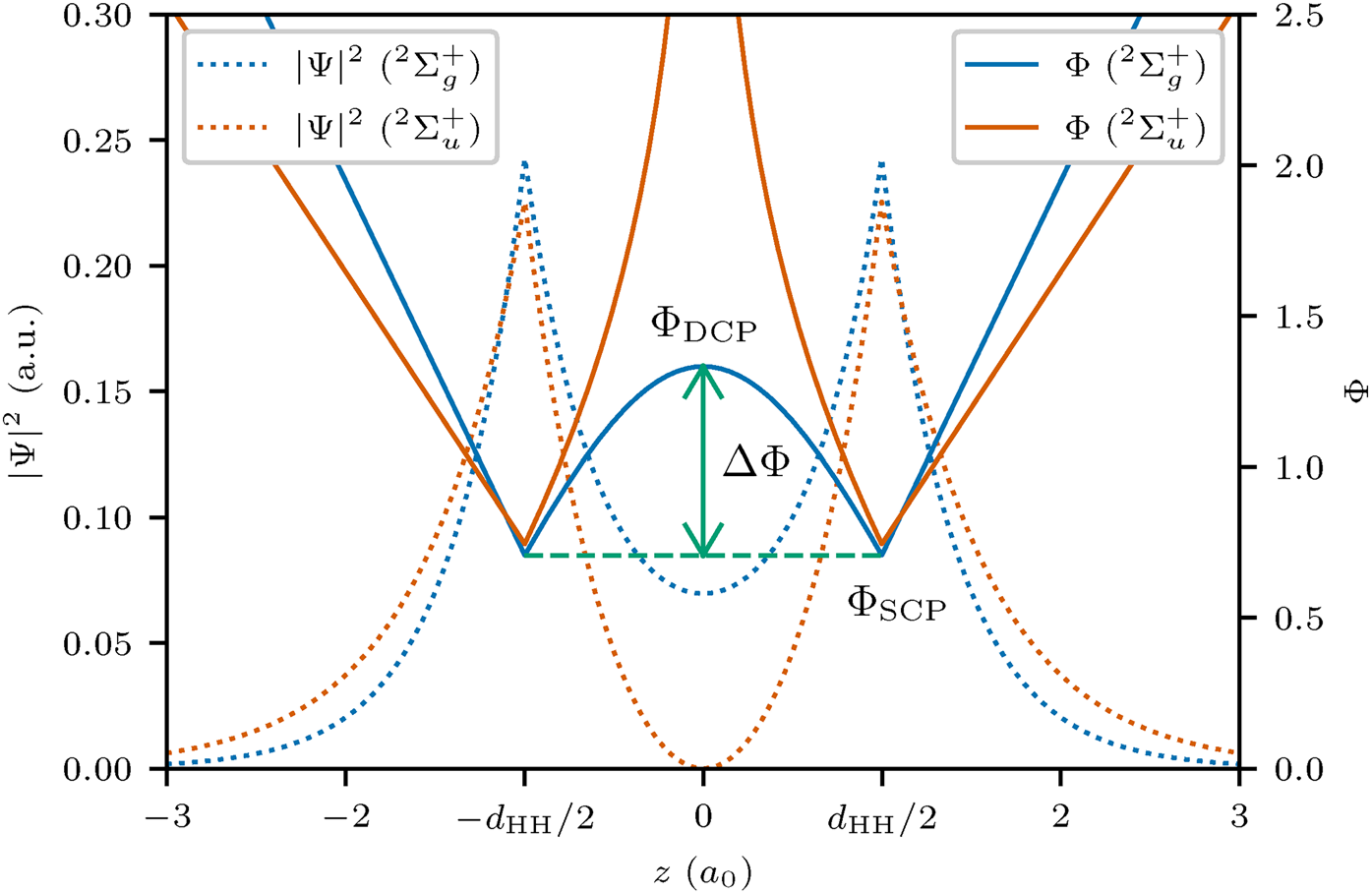
${\left|\Psi \right|}^{2}$ and probability potential Φ for the $H_{2}^{+}$ ground state 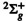 and the anti‐bonding excited state 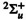 along the z axis connecting the nuclei. Structure critical points (SCP) and delocalization critical point (DCP) are marked. Reproduced from Reference [16] with permission from the Royal Society of Chemistry.

This can be compared to chemical reactions, where the reactants and products would be both minima connected by a saddle point describing a transition state. Recently these barriers have been correlated to covalent bonding in general, and specifically to charge‐shift bonding [16]. Figure 1 shows the SCPs and the DCP for the well‐known example of $H_{2}^{+}$ where ${\left|\Psi \right|}^{2}$ and the electron density coincide. With decreasing bond distance, ΔΦ decreases, and the delocalization increases for the ground state. For the anti‐bonding excited state 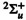, the barrier is infinite due to the vanishing wave function at the midpoint of the nuclei.

## 2 Methodology

The wave functions used for all molecules are Jastrow‐correlated Slater determinants, composed of Kohn Sham orbitals from PBE0 [17] calculations. The Kohn Sham orbitals from different exchange correlation functionals affect the results of PDA only marginally. For the molecular geometries, the experimental geometries from the NIST Computational Chemistry Comparison and Benchmark Database [18] were selected. The wave functions for the carbon and nitrogen atoms are Jastrow correlated linear combinations of Slater determinants from full‐valence CASSCF calculations.

As in previous work, correlated wavefunctions are employed. Hartree‐Fock wavefunctions show maxima of ${\left|\Psi \right|}^{2}$ having opposite spin electrons at the same position which is unphysical and due to the lack of electron correlation. Electron correlation is accounted for with simple Jastrow factors. For all wave functions, a generic sm555 Jastrow correlation‐function [19, 20] was used and optimized with the *amolqc* software [21]. All calculations were performed using the all electron triple zeta Slater‐type basis set TZPae [22]. All DFT and CASSCF calculations were performed using *orca* [23, 24, 25]. For using the STO basis set in orca, each basis function was expanded into 14 Gaussian functions [26, 27].

The probability density analysis was performed with *amolqc* and *inPsights* [28]. The weights of the structures were obtained, by first generating a sample of ${\left|\Psi \right|}^{2}$ using the Metropolis‐Hastings algorithm. By minimizing Φ of the sample points with the steepest descent and the L‐BFGS methods [29], the maxima of ${\left|\Psi \right|}^{2}$ were obtained. Maxima with the same probability potential were counted and their number divided by the total number of maxima to calculate their PDA weights. For the saddle points search, Newton's method was used. In comparison to the generation of the correlated wave functions, the computation time involved in performing the PDA can be considered small. The scaling of the method is favorable due to its Monte Carlo nature. Each electron arrangement discussed in this work has two core electrons at the nitrogen and carbon nuclei.

## 3 Results and Discussion

### 3.1 Maxima of ${\left|\Psi \right|}^{2}$

In this work, the C—C bond in acetylene and the N—N bond in dinitrogen are revisited as examples for triple bonds and studied in detail with our extended PDA tool set. In Table 1, the SCPs of acetylene are classified and sketched (for actual structures see SI). SCPs **a1**—**a3** correspond to spin variations of three banana or τ bonds, where three spin pairings of the six electrons between the carbon atoms with same color indicating same spin are observed. These maxima would correspond to the maxima of the Hartree–Fock localized molecular orbitals if localization was done without symmetry constraint, for example, with the Foster Boys method. The maxima of a Slater determinant consisting of absolutely non‐overlapping orbitals are simply the union of the orbital maxima. A doubly occupied localized banana orbital has two equivalent maxima. The two electrons occupy with equal probability both maxima or the same. Due to the uncorrelated nature of a single determinant wave function, opposite spin electrons can occupy the same maxima positions. The first three SCPs, denoted ecliptic for the ecliptic arrangement of the two triangles on each side of the bond, have the three lowest values of Φ. Their basins cover together 78% of the system.

**TABLE 1:** Stylistic representation of the maxima of ${\left|\Psi \right|}^{2}$ (SCP) for acetylene. The electrons are represented by red and blue circles, while the carbon and hydrogen cores are described by a large grey and a smaller lighter grey circle, respectively. The probability potential Φ and PDA weight is displayed below each maximum.

Ecliptic		Staggered	
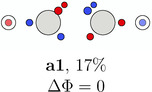	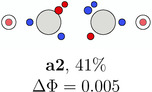	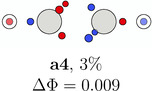	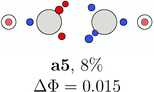
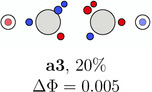		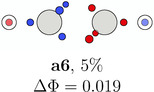	

chair‐like		C—C ionic	
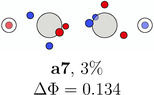	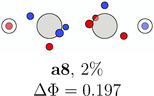	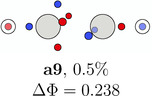	

The next three SCPs, **a4**—**a6** cover further 15% with a staggered orientation of the electron triangles. Note that these SCPs have three same spin electrons on each side while the ecliptic SCPs have one opposite spin electron on each side. The ecliptic and staggered electron arrangements can be rationalized by discussing the Pauli and Coulomb repulsions and thus by the number of same spin electrons at each fragment. In the ecliptic case, minimizing the Pauli repulsion leads to one electron being opposite to the two other same spin electrons on the other side, thus forcing the electrons to form opposite spin pairs. In the staggered case, minimizing Pauli repulsion leads to equilateral triangles on each side and a staggered arrangement to minimizing the Coulomb repulsion between the two triangles. Opposite spin electrons repel each other and need to be forced into pairing.

According to the weights, the ecliptic arrangements seem to be substantially preferred over the staggered ones, but this is mainly a consequence of statistics. When randomly distributing five alpha and five beta electrons on five positions of each C—H group with the only constraint of opposite spin on the C—H bond, three times as many ecliptic arrangements (**a1**—**a3**) as staggered ones (**a4**—**a6**) are obtained. The observed ratio is about five. This deviation fits well to the higher values of Φ for the staggered arrangements corresponding to a lower probability. Note that the staggered SCPs all have a 4 + 1 spin distribution for each C—H group while the ecliptic ones have 3 + 2.

There are further SCPs (**a7**—**a9**) not showing the coupling of six electrons. Their Φ values are considerably larger, and their basins cover only 6% of the system.

In contrast to acetylene, only two SCPs can be identified for dinitrogen, none of which resembles the Lewis structure of a triple bond, see Table 2. In the global maximum **b1**, covering 92% of the system, three same spin electrons around each nucleus form a triangle, and the other two opposite spin electrons a line orthogonal to the plane of the triangle, that is, a distorted trigonal bipyramid. This maximum also has the highest PDA weight. The second SCP **b2**, is described by each atom having four same spin electrons and one electron of opposite spin. These electrons form a tetrahedron with one corner on the bond axis between both atoms. The opposite spin electron is located on the bond axis, but behind the nitrogen atom. Note that for dinitrogen, a random distribution of the electrons would yield combinatorially only a 2:1 preference of **b1** over **b2**.

**TABLE 2:** Stylistic representation of the maxima of ${\left|\Psi \right|}^{2}$ for dinitrogen. The electrons are represented by red and blue circles, while the nitrogen cores are described by a large blue circle. The probability potential Φ and the PDA weight are shown.

Global maximum	Local maximum
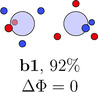	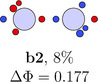

Recently, Menéndez‐Herrero and Martín Pendás have shown that the electron arrangements present in molecules can often be rationalized by combining the electron arrangements found in atoms [30]. Here, the maxima for the lowest states of the nitrogen and carbon atom have been calculated with symmetry‐adapted complete‐active‐space self‐consistent field wavefunctions. In Table 3 the maxima are ordered with increasing energy for both atoms. While the lowest quartet and doublet states for N and the triplet and quintet state of C follow a minimization of Pauli and Coulomb repulsions, the three other states, **n3**, **c2**, **c3**, have planar electron arrangements. In the case of C 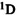, the plane is defined by the two singly occupied 2p orbitals, and the planarity of the N 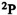 has a similar origin. The plane in C 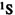 is a correlation effect, without the Jastrow correlation function two opposite spin electrons occupy the same position, and the axis combining both pairs rotates freely (due to identical occupancies of $p_{x}$, $p_{y}$ and $p_{z}$ in C 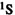).

**TABLE 3:** Stylistic representation of the maxima of ${\left|\Psi \right|}^{2}$ for a Jastrow correlated Slater determinant for $p^{2}$ and $p^{3}$ Russell–Saunders states of C and N. The electrons are represented by red and blue circles, while the carbon and nitrogen atoms are described by large grey and blue circles. The electrons in maxima **n3**, **c2**, and **c3** are located in the same plane.

N 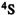	N 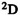	N 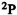	C 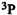	C 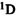	C 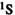	C 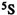
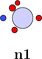	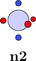	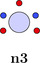	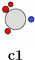	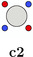	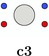	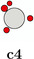

Upon comparison of the SCPs for the nitrogen atom with the arrangements in dinitrogen, it is evident that SCP **b1** can be thought as composed of twice the 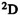 state and SCP **b2** of twice the 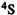 state. While the electron positions for the 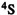 nitrogen atom match the arrangement found in SCP **b2** exactly, the positions for the 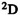 state are distorted in dinitrogen due to the Pauli repulsion. The electron arrangements for the 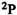 state cannot be found in the SCPs of dinitrogen, but only in a DCP (see SI). With elongation of the N—N bond, the weight of **b2** increases and reaches 100% at dissociation as 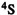 is the ground state of N. The SCPs found in acetylene correspond to the arrangements found for the 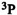 and 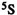 state in the carbon atom (Table 3 **c1** and **c4**). All the electron arrangements for the other states in the carbon atom cannot be identified in the acetylene molecule.

Why do the electron arrangements in dinitrogen differ from those in acetylene and thus from the Lewis structure? Let us start with a staggered SCP (**a4**—**a6**) of acetylene and replace the CH cores with N. This allows the C—H bond electrons to relax and to form same spin tetrahedra around each N as dictated by Pauli repulsion. The SCP **b2** results then from maximizing Coulomb repulsion. The global maximum **b1** arises similarly from relaxing the trigonal bipyramids. Due to the short bond distance the Coulomb repulsion between the two same spin triangles can be optimized by rotation away from the bond axis. The rotation angle is strongly dependent not only on the bond distance, but also on correlation factor. Therefore, the arrangement of three pairs in acetylene is due to forcing one vertex of each carbon valence electron tetrahedron towards the attracting hydrogen nucleus. This is consistent with the orientation of the carbon tetrahedra in ethane and ethene, where a single bond and a double bond motif arise naturally, see Table 4.

**TABLE 4:** Stylistic representation of the global maxima of ${\left|\Psi \right|}^{2}$ for a Jastrow correlated Slater determinant for ethane (**e1**), ethene (**f1**), and acetylene (**a1**). The electrons are represented by red and blue circles, while the carbon and hydrogen atoms are described by a large grey and a smaller lighter grey circle. The PDA weight is displayed below each maximum.

Ethane	Ethene	Acetylene
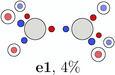	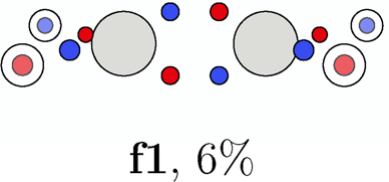	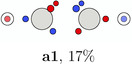

**FIGURE 2:**
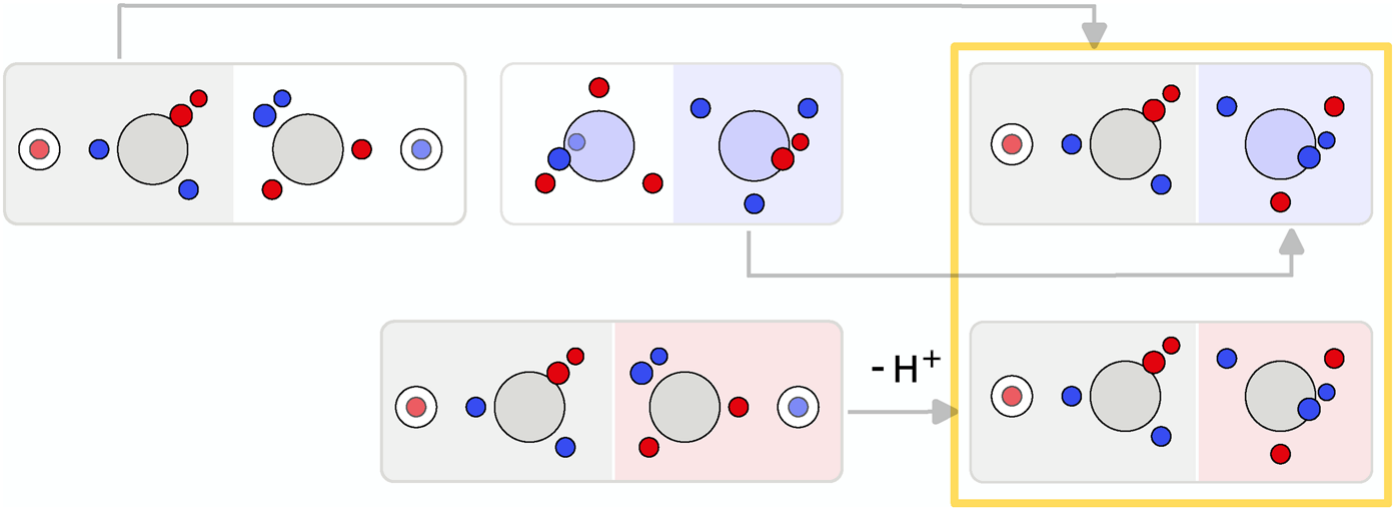
Schematic representation of the electron arrangements in the global maxima of HCN and ${\mathrm{HCC}}^{-}$. The electrons are represented by red and blue circles, while the nitrogen, carbon, and hydrogen atoms are described by a large blue, large grey, and a smaller lighter grey circle.

To further study the influence of the hydrogen atoms, HCN and deprotonated acetylene (HCC^−^) were calculated. The resulting SCPs in HCN and HCC^−^ are composed of combinations of the SCPs of the CH fragment and the N atom (Figure 2). Upon removing one of the H atoms in acetylene, the remaining negatively charged C atom is equivalent to a nitrogen atom except for its nuclear charge. This leaves a nitrogen‐like atom with five valence electrons and no more C—H bond to locate one of them on. As a result the electron arrangements in HCC^−^ are almost identical to the SCPs in HCN. The smaller nuclear charge only leads to more ionic electron arrangements for HCC^−^ compared to HCN.

Concluding, there are two contributing factors determining the electron positions maximizing ${\left|\Psi \right|}^{2}$ in dinitrogen and acetylene. First, there are the SCPs found in the atomic calculations that, when combined, result in the electron arrangements found in N_2_ and C_2_H_2_. Second, the hydrogen atoms play a significant role in the orientation of the atomic SCP, resulting in a Lewis structure like motif for acetylene but not for dinitrogen.

### 3.2 Maximum Probability Paths

Both dinitrogen and acetylene have very strong bonds. While acetylene is characterized by a triple bond motif with three spin‐coupled electron pairs as shown above, dinitrogen is not. How can the stabilization of dinitrogen be retrieved from the probability density? The stabilization of a spin‐coupled pair is due to the electron delocalization given by the exchange of the opposite spin electrons, in connection with the potential energy. Low probability barriers ΔΦ correspond to strong delocalization along this path and high probability barriers to little delocalization [15, 16]. Note that strongly stretched single bonds still shows full spin coupling, but a high probability barrier ΔΦ and correspondingly only a small bond dissociation energy. Therefore, the delocalization critical points (DCP) and the corresponding probability barriers for both dinitrogen and acetylene were investigated.

As a result of the antisymmetry of the wave function, two SCPs where a pair of same spin electrons is exchanged cannot be connected by a maximum probability path. This exchange would require the sign of the wave function to change, therefore the probability density would become zero at some point along the exchange, resulting in an infinitely large probability barrier. However, a simultaneous exchange of three same spin electrons is possible as this permutation has a positive sign in the antisymmetrizer. Moreover, pairwise exchanges of opposite spin electrons do not change the sign of wave function, due to the different spins of the electrons. Additional probability barriers connect different maxima structures such as ecliptic and staggered or ecliptic and chair‐like for acetylene (Table 1). However, these barriers are not discussed in this work.

In acetylene, the SCP with the largest weight, **a2**, has one triangle composed of same spin electrons at each fragment. A rotation of these triangles results in an electron arrangement where only the indices of the electrons have changed. Therefore, the starting and ending SCPs have the same probability potential. For dinitrogen, the SCP with the largest weight also contains these triangles, which again can rotate. Although the triangles for each atom in dinitrogen cannot rotate independently, they can both rotate in the same or in the opposite direction simultaneously (See Data S1). Therefore, by combining these two paths the result of the rotation of a single triangle can still be obtained. When comparing the probability barriers of these rotations, the barrier in dinitrogen is an order of magnitude smaller than that in acetylene.

Again this can be explained by electrostatics and Pauli repulsion: One of the electrons in acetylene lies on the C—H bond. Due to the favorable electron nucleus attraction, the motion of this electron is hindered. In dinitrogen, the lack hydrogen atoms lowers the barrier significantly and increases the delocalization. This can be further corroborated by investigating the rotation of these triangles in ethene and ethane. The probability barrier correlates with the number of electrons located on a C—H bond that are involved in the rotation. While it is largest for ethane, where two electrons are located on such bonds, for ethene and acetylene it is smaller, because only one electron is located on such a bond (Table 5). Thus, the electrons forming these triangles are more delocalized in dinitrogen than in acetylene. Moreover, it can also be seen that the delocalization of the bond electrons grows larger for triple bonds compared to single or double bonds.

**TABLE 5:** Stylistic representation of the rotations of triangles composed of three same spin electrons (represented by blue and red circles) in ethane, ethene, acetylene, and dinitrogen. The nitrogen, carbon, and hydrogen atoms are shown as blue, grey, and light grey circles. The probability barrier of each exchange is shown below the structures and reference to one of the starting maxima.

Ethane	Ethene	Acetylene	Dinitrogen
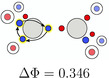	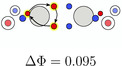	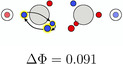	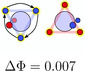

The triangular rotation contributes to the delocalization of the electrons in the molecule, but not directly to the bond stabilization through electron exchange between the fragments. Such paths, describing the exchange of two opposite spin electrons between the two fragments, were also found. Although there are multiple possibilities, only two, having the lowest barrier, will be discussed here, see Table 6. The probability barriers in dinitrogen are significantly smaller than in acetylene. Upon closer inspection of the saddle point **B1** in dinitrogen, it is apparent that after the exchange two new triangles consisting of same spin electrons are formed. Therefore, through combinations of this exchange and rotations of these triangles, every electron can move across the whole molecule with very small probability barriers. Thus, every valence electron is involved in the N—N bonding. In acetylene, a similar exchange exists (**A1**), connecting the SCPs **a1** and **a3**. Here, electrons 2 and 6 move to the other C atom. This exchange is best described by simultaneous rotation of the up spin triangle 2‐3‐7 and the down spin triangle 4‐5‐6. However, the probability barrier is more than twice as large as the barrier in dinitrogen. While the barrier in acetylene is larger, it is (almost) fourfold degenerate. This multiplicity balances to some extent the larger delocalization in dinitrogen because of the lower barrier.

**TABLE 6:** Stylistic representation of the starting and ending maxima and the connecting saddle point of opposite spin electron (red and blue circles) exchanges between both fragments in dinitrogen and acetylene with their probability barrier. The nitrogen, carbon, and hydrogen atoms are shown as blue, grey, and light grey circles. The black arrows displayed for the starting maximum summarize the electron exchange. The red and blue arrows for the saddle point represent the eigenvector corresponding to a negative eigenvalue of the Hessian.

Starting maximum	Saddle point	Ending maximum
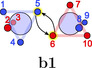	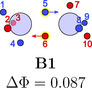	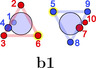
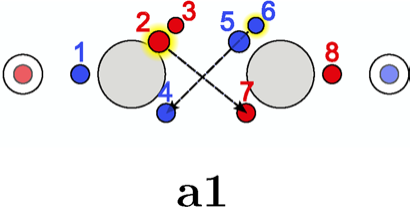	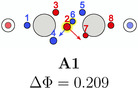	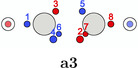

This trend applies to all exchanges in both molecules. Thus, the electrons in dinitrogen are more likely to exchange their positions, they are more delocalized compared to acetylene. While no connection to the energetics of these systems has been made, it is clear that this difference correlates with the different chemical behaviors of both molecules.

## 4 Conclusions

Dinitrogen and acetylene were analyzed with probability density analysis. While the electron arrangements in acetylene resemble the Lewis structure, the arrangements in dinitrogen do not.

It was found that the SCPs in dinitrogen and in acetylene are mostly composed of the electron arrangements found for the isolated nitrogen and carbon atoms. The orientation of these SCPs in the molecules can be explained by electrostatics and Pauli repulsion, and more precisely by the presence of hydrogen atoms. Due to the C—H bond in acetylene, the four valence electrons of the carbon atom are oriented in a way that the resulting electron arrangement resembles the Lewis structure. The same explanation applies for the electron positions maximizing ${\left|\Psi \right|}^{2}$ in ethane and ethene. For dinitrogen, no combination or orientation of one of the SCPs found in the isolated atoms results in a triple bond motif.

The analysis of the maximum probability paths reveals that the probability barriers for electron exchanges are significantly lower in dinitrogen compared to acetylene, leading to greater electron delocalization in dinitrogen. This difference in probability barriers is attributed to the absence of hydrogen atoms in dinitrogen, which reduces the electrostatic hindrance present in acetylene. This is confirmed by the even larger probability barriers in ethane and ethene. Therefore, the delocalization of the bond electrons is increased for multiple bonds and thus the stabilization. Moreover, most of these exchanges consist of rotating a triangle formed by three same spin electrons. Contrary to the exchange of two same spin electrons, these rotations are allowed by the antisymmetry principle.

This shows, that an investigation of molecules with PDA gives insights beyond the Lewis picture and finds significant differences for dinitrogen and acetylene correlating with their drastically different chemical behaviors.

## Supporting information


**Data S1:** Supporting Information.

## Data Availability

The data that support the findings of this study are openly available in the Open Science Framework at http://doi.org/10.17605/OSF.IO/DJTC9.
