# Supplementary material for: Probability Density Analysis Reveals Substantial Differences Between the Dinitrogen and Acetylene Triple Bonds
Source: J Comput Chem. 2025 Feb 3;46(4):e70037. doi: 10.1002/jcc.70037 (PMC11791126; doi:10.1002/jcc.70037)
Supplement: Supplementary file 1 — Data S1: Supporting Information. [file JCC-46-0-s001.pdf]

Supplementary Information: Probability Density Analysis reveals substantial differences between the dinitrogen and acetylene triple bonds

Michel V. Heinz[*], Nicole Maser[*], Emma Gorgas[*], Arne Lüchow[*]

August 2024

[*]Institute of Physical Chemistry, RWTH Aachen University

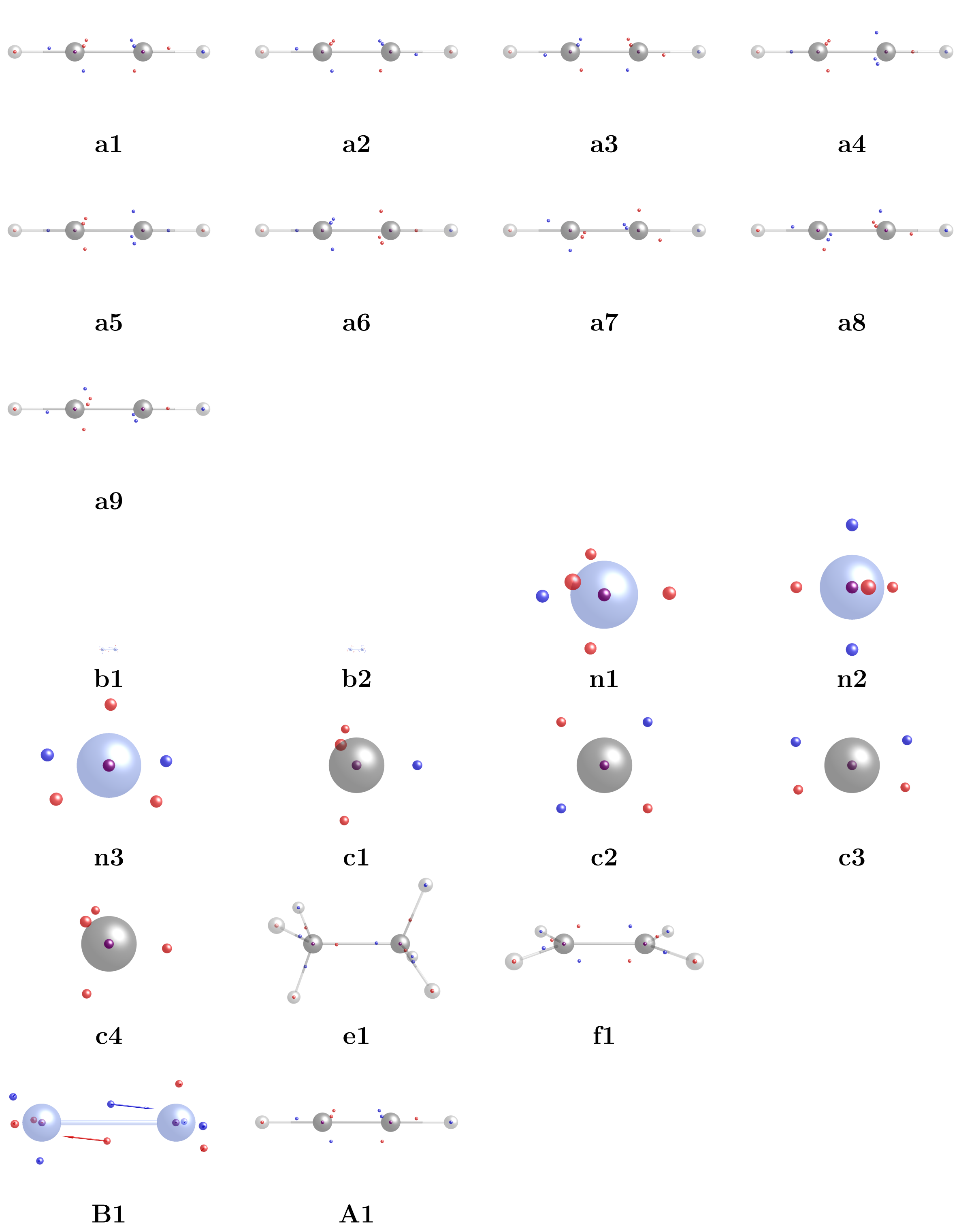

Table SI 1: Electron positions (blue and rinked spheres) of critical points of $|\Psi|^2$ for all the stylistic representation.

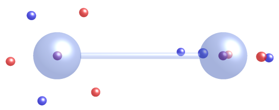
**B4**

Table SI 2: DCP in dinitrogen corresponding to the electron arrangement found for the ^{2}P state of the nitrogen atom.

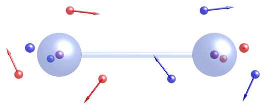	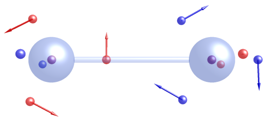
**B2**	**B3**

Table SI 3: DCPs representing the rotation in the same and opposite direction of the same spin electron triangles in dinitrogen.
